# A haplotype‐based approach to explore genotype‐by‐environment interactions for barley yield

**DOI:** 10.1002/tpg2.70310

**Published:** 2026-09-29

**Authors:** Stephanie M. Brunner, Zachary Aldiss, Samir Alahmad, Madhav Pandit, Silvina Baraibar, Dini Ganesalingam, David Moody, Lee Hickey, Kai P. Voss‐Fels, Hannah Robinson

**Affiliations:** ^1^ Queensland Alliance for Agriculture and Food Innovation The University of Queensland St Lucia Queensland Australia; ^2^ InterGrain Pty Ltd Perth Western Australia Australia; ^3^ Department of Plant Breeding Hochschule Geisenheim University Geisenheim Germany

## Abstract

Climate change poses a significant threat to cereal production, making it increasingly important to understand the role of genotype‐by‐environment interactions (GEIs) in crops. Traditional breeding approaches consider GEIs at a whole genotype level missing valuable breeding potential that can be dissected by exploring this relationship at a chromosome segment level. A robust commercial dataset consisting of 961 spring barley (*Hordeum vulgare* L.) genotypes grown across 12 sites was used to explore these questions and to co‐locate haplotypes under GEIs with key physiology genes to better understand the drivers of GEIs. A genomic best linear unbiased prediction model for barley yield was fitted to the data, where the GEI term had a factor analytic 4 (FA4) variance structure. Using the first three factor loadings from the FA4 model, the 12 sites were grouped into four environmental clusters. The barley single nucleotide polymorphisms were partitioned into 1741 haploblocks based on linkage disequilibrium and the local genomic estimated breeding values were calculated for each haplotype in each environmental cluster. The GEIs of each haploblock across the environmental clusters were explored, and those under large GEIs were identified across several chromosomes and found to vastly vary in size. Further analysis of a few key haploblocks revealed that for some blocks the best haplotypes were similar across clusters, but for others they were vastly different. This study provides a novel framework for investigating GEIs at a haplotype level, which can be utilized in a range of breeding applications across traits and crops.

## Plain Language Summary

Climate change presents a growing challenge to cereal production, highlighting the need to better understand genotype‐by‐environment interactions (GEIs). Conventional breeding typically evaluates GEIs at the whole‐genotype level, which can obscure important variation within the genome. In this study, 961 spring barley genotypes grown across 12 environments were analyzed to investigate GEIs at the haplotype level. A genomic best linear unbiased prediction model with a factor analytic structure was used to characterize GEIs and group environments into four clusters. The genome was partitioned into haplotype blocks based on linkage disequilibrium, and local genomic estimated breeding values were calculated for each block within each environmental cluster. This enabled the identification of genomic regions exhibiting strong GEIs. Results showed that some haplotypes were consistently favorable across environments, while others displayed environment‐specific effects. This haplotype‐based framework provides a more resolved understanding of GEIs and offers practical opportunities to enhance selection strategies in crop breeding.

**Abbreviations**
AIC: Akaike information criterion
BIC: Bayesian information criterion
EV%: percentage of error variance
FA: factor analytic
GBLUP: genomic best linear unbiased prediction
GEI: genotype‐by‐environment interaction
LD: linkage disequilibrium
LGEBV: local genomic estimated breeding value
PC: principal component
QTL: quantitative trait loci
SNP: single nucleotide polymorphism

## 1 INTRODUCTION

Climate change is causing increasingly rapid changes to environmental conditions globally (Head et al., 2014). For instance, Australia has already seen temperature increases and altered rainfall patterns with drought becoming more prevalent in several key growing regions (Cook et al., 2018; Hughes, 2003). Cereal crops are especially sensitive to changes in weather; recent modeling shows climate change has caused Australian wheat yield potential to decline by approximately 12% between 1990 and 2023 (Lawes et al., 2025). The sensitivity of growing systems to environmental conditions makes understanding the role of environment on genetic performance vital to maintaining cereal yields for both the Australian and global markets.

The goal of many cereal breeders is to identify consistently high‐performing varieties that are stable across environmental conditions. Breeding for trait stability across environments has been successful in traits such as disease resistance, which in many cases are influenced by a small number of loci and can show relatively consistent expression across environments (Mahesha et al., 2022). In contrast, achieving stability in complex traits like yield is far more challenging, whereby highly stable varieties are not necessarily the highest performing in any given environment, yet they remain valuable because their performance is more predictable (Pour‐Aboughadareh et al., 2023). Genotype‐by‐environment interactions (GEIs), the differential responses of genotypes to different environmental conditions, remain a central challenge in breeding for stability, particularly under increasing environmental variability (Cooper & Messina, 2021; Xiong et al., 2022). When breeding for stability, it is important to consider the range of environments within a target region, as stability refers to predictable performance across the variable conditions experienced within that region (Chapman et al., 1997). While breeding has improved stability for traits such as yield, there is the potential to leverage GEI through enviromics and targeted selection for common environment types in order to enhance adaptation (Cooper & Messina, 2021). Improved characterization and prediction of environmental conditions offer opportunities to better understand and utilize the environmental dimension of GEI when developing future varieties.

GEIs can be modeled through a range of approaches, including reaction norms, machine learning, and Bayesian approaches (Fernandes et al., 2024; Waters et al., 2023; Zhang et al., 2014). Breeding programs widely use linear mixed models to explore GEIs in a linear framework. Incorporating GEIs into linear mixed models as a random effect has been found to increase power to detect significant quantitative trait loci (QTL) and generate better breeding decisions (Erbe et al., 2022; Tiret et al., 1993). However, it is the approach to fitting the variance of this random term that determines its overall effectiveness in capturing this relationship. Falconer (1952) was the first to propose fitting these interactions as separate but correlated traits for each environment resulting in the development of several variance structures, which are still utilized today. Simplistic variance structures such as CorV and CorH, which assume heterogeneous and homogeneous variance, respectively, have been used to account for this shared variance between GEI terms (Falconer, 1952). An unstructured variance‐covariance structure can be specified by estimating a full variance‐covariance matrix, comprising environment‐specific variances and pairwise covariances between environments. However, this approach is computationally demanding, particularly when the number of environments is large. The factor analytic (FA) model provides a flexible variance‐covariance structure by representing the covariance between environments through a set of latent factors (Smith et al., 2001). Each factor captures an independent dimension of environmental variation, with environment‐specific loadings describing how strongly each environment relates to these underlying dimensions. The FA model has been used in several studies for traits such as disease resistance, yield, and stay‐green in wheat (*Triticum aestivum* L.), barley (*Hordeum vulgare* L.), and maize (*Zea mays* L.), among others, to fit the GEI term (Brunner et al., 2024; Chaves et al., 2023; Ferrante et al., 2021; Pandit et al., 2026; Vo Van‐Zivkovic et al., 2025). An additional benefit of the FA model is that the rotated environment‐specific loadings can be utilized to define interaction classes (iClasses), which can be used to cluster environments (Smith et al., 2021). Defining of environmental clusters serves as an efficient means by which to group environments to explore the GEI between environment types. Well‐defined environmental clusters are characterized by minimal crossover GEI within clusters but crossover GEI between clusters, such that breeding decisions can be made within each cluster (Russell et al., 2003).

GEI in cereals are typically examined at the whole‐genotype level by comparing the performance of varieties across environments. While informative, this approach lacks the resolution needed to identify the specific genomic regions underlying differential environmental responses, thereby limiting its utility for targeted breeding applications. Single nucleotide polymorphism (SNP)‐based GEI analyses (Boer et al., 2007; Moreau et al., 2004) offer finer resolution but treat markers independently, ignoring the effects of linkage and recombination, factors that ultimately determine which allele combinations can be transmitted to the next generation. Haplotype‐based approaches provide a biologically meaningful intermediate scale by capturing blocks of linked alleles that are inherited together. This makes haplotypes a more realistic unit for understanding and exploiting GEI in breeding programs, as they reflect the genomic segments that can be reliably inherited and selected. Building on earlier work demonstrating the utility of local genomic estimated breeding value (LGEBV) or haplotype‐based prediction frameworks (Voss‐Fels et al., 2019), recent studies have shown that variation in haplotype effects across genomic blocks (haploblocks) can reveal regions associated with trait expression and stability (Aldiss et al., 2025; Brunner et al., 2024; Pandit et al., 2026; Vo Van‐Zivkovic et al., 2025). Haplotype‐level evaluation therefore provides a biologically meaningful and operationally useful resolution for identifying genomic regions underlying GEIs.

Using a combination of modern statistical approaches, this study proposes a new crop‐agnostic methodological framework for exploring GEI at the haplotype level for application in breeding programs. In contrast to existing haplotype‐level GEI frameworks, which employ a two‐stage approach, the method presented here uses a one‐stage model in which haplotype effects are back‐solved directly, avoiding the propagation of error between stages and making fuller use of the information in the data. Barley provides an effective case study for evaluating the framework due to its well‐documented environmental responsiveness (Macholdt et al., 2020), extensive multi‐environment trial resources, and well‐characterized and annotated genome. The framework provides information on GEI stability and variability at a haplotype level and specifically aims to (1) compare and contrast high‐variance haploblocks across environmental clusters, (2) identify haploblocks exhibiting GEI for yield, and (3) explore how haplotype effects change across environmental clusters at key haploblocks.

## 2 MATERIALS AND METHODS

### 2.1 Yield data

A total of 961 barley genotypes were assessed for yield performance in 12 field trials held across regions of Western Australia and Queensland during the 2020, 2021, 2022, and 2024 growing seasons (Table 1) with a moderate level of genotype concurrence between each pair of trials (Table S1). Germplasm was provided by Australian Breeding company InterGrain Pty Ltd and is representative of Australian barley breeding from the last decade. Queensland Trials (22GAT and 24GAT) were managed by the University of Queensland and sown as plots measuring 2 by 6 m, cut back to a final area of 10 m^2^, with partial replication in a randomized block design. Western Australian trials were managed by InterGrain Pty Ltd and were 5 m^2^ plots with partial replication. Yield was converted to tonnes per hectare (t/ha) prior to analysis. All trials were conducted under rainfed conditions, with insect and weed management applied as necessary.

**TABLE 1:** Trial data for 12 trial sites with broad‐sense heritability for yield.

Trial	Region	Sowing	Harvest	Latitude	Longitude	Site mean yield (t/ha)	*H* ^2^
20YRK	WA	7/05/2020	19/11/2020	−32.03	116.86	5.14	0.67
20MRA	WA	22/05/2020	6/11/2020	−30.63	116.07	2.52	0.66
21CGN	WA	22/05/2021	17/11/2021	−32.36	117.94	4.61	0.66
21DND	WA	12/05/2021	16/11/2021	−30.75	115.66	4.26	0.67
21SCW	WA	14/05/2021	21/11/2021	−33.44	121.59	5.52	0.76
21YRK	WA	17/05/2021	30/11/2021	−32.03	116.83	4.64	0.69
22GAT	QLD	16/06/2022	10/11/2022	−27.54	152.35	4.73	0.34
22CGN	WA	26/05/2022	23/11/2022	−32.38	117.94	4.59	0.50
22DND	WA	5/05/2022	14/11/2022	−30.65	115.78	4.37	0.68
22SCW	WA	9/05/2022	26/11/2022	−33.26	121.37	4.95	0.68
22YRK	WA	19/05/2022	14/11/2022	−32.02	116.86	4.54	0.58
24GAT	QLD	21/05/2024	25/10/2024	−27.54	152.32	3.00	0.83

Abbreviations: QLD, Queensland; WA, Western Australia.

### 2.2 Genotype data

The barley panel was genotyped using wheat barley 40K v1.0 BeadChip returning 12,548 high‐quality SNPs for barley (Keeble‐Gagnere et al., 2021). Prior to downstream analysis, these markers were curated to remove markers with >10% heterozygosity and a minor allele frequency ≤0.05 resulting in 6599 SNPs. Principal component (PC) analysis was conducted on the curated marker data utilizing a genetic distance matrix calculated with Rogers’ distance (Rogers, 1972) to identify clusters of individuals from similar genetic backgrounds. The dendrogram of genotype relatedness was used to determine appropriate number of clusters present in the panel.

### 2.3 Multi‐environment genomic best linear unbiased prediction model with GEI

To model GEI and estimate SNP effects within a single framework, a genomic best linear unbiased prediction (GBLUP) model was applied to the raw yield data of each environment (Equation 1). Preliminary analysis involved the removal of outliers.

GBLUP equation

(1) $$\begin{array}{ccc} y & = & X\beta +Z_{g}u_{g}+Z_{g2}u_{g2}+Z_{r}r\\ & & +\;Z_{c}c+\varepsilon ,\;\varepsilon ∼N\left(0,\;{E\sigma }_{e}^{2}\right) \end{array}$$

where y is the vector of observed phenotypic values, β is vector of fixed environmental effects with corresponding design matrix X. The term $u_{g}$ represents the random additive genetic effect and $Z_{g}$ the corresponding design matrix. The term $Z_{g2}u_{g2}$ represents a residual genetic effect not captured by the additive genomic relationship matrix (GRM) and its associated design matrix. The terms $Z_{c}c$ and $Z_{r}r$ represent the random effects associated with row and column for each environment. The residual term ε was modeled as *ε* ∼ *N*(0, *Eσ_e_* ^2^), where *ε* follows a first‐order autoregressive by first‐order autoregressive (AR1 × AR1) variance‐covariance structure, accounting for spatial correlation along both rows and columns of the field layout.

This model was fitted using ASReml‐R (version 4.2) with the environment (year–site combination) as a fixed effect, and all other effects fitted as random. The genomic relationship matrix was calculated using the R package “AGHmatrix” using the Van Raden method (Amadeu et al., 2023; VanRaden, 2008).

A range of variance‐covariance structures were evaluated for the GEI term, including diagonal and correlation‐based models assuming homogeneous and heterogeneous variances. An unstructured variance‐covariance matrix, which estimates all variances and pairwise covariances independently, is generally the most flexible approach; however, it was computationally infeasible for this dataset. Therefore, an FA model was used, which approximates the unstructured matrix by modeling the covariance among environments using a reduced number of latent factors (Piepho, 1998; Smith et al., 2015). The optimum model was identified by several model statistics (see below model statistics). It was found that an FA4 model was the best fit for the variance of the interaction between environment and additive genetic component.

FA4 model

(2) $$\begin{array}{ccc} u_{g} & = & \left(\lambda_{1}⊗\;I_{m}\right)\;f_{1}+\left(\lambda_{2}⊗\;I_{m}\right)f_{2}+\left(\lambda_{3}⊗\;I_{m}\right)f_{3}\\ & & +\left(\lambda_{4}⊗\;I_{m}\right)f_{4}+\delta \end{array}$$

where λ_i_ represents the vector of loadings for the *i*th factor, describing how each environment relates to that latent factor, I_m_ is an identity matrix of appropriate dimension, *f_i_* are the latent factors capturing independent dimensions of genetic covariance across environments, and δ is the vector of residual effects (Smith et al., 2001). The residual GEI term, capturing genetic variation not explained by the additive component, was modeled using a FA structure of order 2 (FA2).

The GBLUP equation was rearranged to estimate marker effects (α) as shown in Equation (3):

Marker effects from GBLUPs

(3) $$\alpha ={\left(M^{T}\left(MM^{T}\right)\;\right)}^{-1}\;\hat{g}$$

where M is the genotype marker matrix (with dimensions number of genotypes individuals by number of markers) and $\hat{g}$ is the vector of GBLUPs obtained from Equation 2. Marker effects were estimated with respect to the reference allele.

To assess the reliability of the estimated genetic correlations, a reduced model with an unstructured variance‐covariance matrix for the additive GEI term was fitted. Due to computational constraints, the residual GEI component ($Z_{g2}u_{g2}$), capturing genetic variation not explained by the additive term, was excluded. The model included environment as a fixed effect, with additive GEI, residual genetic effects, and row and column effects fitted as random terms.

### 2.4 Multi‐environment GBLUP model without GEI

To determine the appropriateness of using this dataset to explore GEI, a multi‐environment model with no GEI term or variance structure was fitted to the data. The form of the model was identical to Equation (1), but the GEI terms $u_{g1}$ and $u_{g2}$ were excluded and replaced with genetic main effect terms $g∼N(0,\sigma^{2}A)$ and $g_{1}∼N\left(0,\sigma^{2}\right)$ where *A* is the GRM and $g_{1}$ captures the residual genetic variation not explained by *A*. GBLUPs and marker effects were derived as described in Equations (1) and (3).

### 2.5 Model fit statistics

A range of model statistics were utilized to assess variance structures for GEI component as well as to compare the GEI and non‐GEI models. Widely utilized model statistics were chosen for initial comparison, Loglikelihood (Logl), Akaike information criterion (AIC), and Bayesian information criterion (BIC) (Akaike, 1974; Schwarz, 1978). In addition to these statistics, the percentage of error variance (EV%), modeling efficiency (EF), and percentage of genetic variance accounted for were calculated for each model. A likelihood ratio test was conducted using the ASReml‐R function “lrt” on each GEI model to test for statistically significant model improvement and ensure selection of the most parsimonious model.

The EV% is indicative of the amount of error present in the model, and as such, lower values are considered favorable and is calculated by Equation (4).

Percentage of error variance

(4) $$\mathrm{EV}\%=\frac{\mathrm{MSE}}{\Sigma \sigma^{2}}\times 100$$

where σ represents the total variance and MSE is the mean square error as calculated by $\frac{1}{n}\Sigma {\left(Y_{i}-{\hat{Y}}_{i}\right)}^{2}$ where *n* is the number of observations, *Y* is the observed value, and $\hat{Y}$ the predicted value from the model.

The modeling efficiency (Equation 5) is a goodness‐of‐fit statistic, similar to the widely used *R* ^2^ value, however it has been found to be better suited to complex models with a range of negative infinity to 1 where 1 indicates a perfect model fit (Mayer & Butler, 1993).

Modeling efficiency

(5) $$\mathrm{EF}=1-\frac{\Sigma {\left(Y_{i}-{\hat{Y}}_{i}\right)}^{2}}{\Sigma {\left(Y_{i}-{\bar{Y}}_{i}\right)}^{2}}$$

where $\bar{Y}$ represents the mean of the observed values. Such the modeling efficiency is equal to one minus the ratio of the sum of squared prediction errors to the total observed variation.

### 2.6 iClasses for environmental clustering

An FA model utilizes a set of “factors” determined by identifying common variation across environments. The polarity of loading for each of the rotated factors allows the determination of iClasses, which are groups of environments with minimal crossover GEI (Smith et al., 2015). iClasses were defined using the first three factor loadings from the FA4 model and were used to create environmental clusters (CL1, CL2, CL3, and CL4). Further validation of the FA4 variance structure used to define iClasses was conducted through the use of a simplified model with an unstructured variance structure applied to the GEI term allowing for the independent calculation of variances and covariances between the sites. Marker effects were estimated for each environmental cluster by back‐solving from the corresponding GBLUPs using Equation (3). This approach enables comparison of haplotype effects across contrasting environmental conditions.

### 2.7 Local genomic estimated breeding values

Haploblocks were defined per the methodology in Shaffer et al. (2025) based on the linkage disequilibrium of the 6599 SNPs, with an linkage disequilibrium (LD) threshold of 0.6 and a tolerance of 3. Tolerance refers to the number of markers included in the haploblock that do not reach the required threshold. These parameters were selected due to the level of LD in the population (average LD between adjacent markers ∼0.53) and to limit any misalignment issues with the Morex Version 3 genome (Mascher et al., 2021). The analysis was conducted using the R package Selection Tools (Selection tools developers, 2023), though the same methodology can be implemented in HapSelect (Brunner, 2026; Shaffer et al., 2026). A resulting average of ∼3.8 markers per block represents a reduction in dimensionality practical to breeding outcomes but without a loss of resolution. This resulted in a final number of 1741 haploblocks across the seven chromosomes ranging in size from 1 to 185 SNPs (Table S2).

Cluster‐specific haplotype effects were calculated as the sum of marker effects within each haploblock, based on marker effects back‐solved from the cluster‐specific GBLUPs. The variance of haplotype effects within each haploblock was then used to identify genomic regions contributing to variation in yield across and within environmental clusters (Table S2).

### 2.8 Local genotype‐by‐environment interactions

Local genotype‐by‐environment interactions (LGEI) was calculated as a measure of the GEI specific to each haploblock across the clusters. This statistic was designed to assign the highest values to the blocks with the greatest GEI at a scale relevant to breeding. The statistic is the sum of the product of the variance of each block across all environments and the mean summed squared pairwise differences among environmental cluster effects for each haplotype (Equation 6), capturing both the blocks’ overall importance for yield and GEI.

LGEI calculation

(6) $$\mathrm{LGE}I_{i}=\mathrm{Va}r_{\mathrm{al}l_{i}\;}\;\times \;\frac{1}{n}\;\sum_{j\;=1}^{n_{i}}\sum_{p<q}{\left(CL_{\mathrm{pj}}-CL_{\mathrm{qj}}\right)}^{2}$$

where $\mathrm{Va}r_{\mathrm{al}l_{i}\;}$ is the variance of block *i* as across all environments, $n_{i}$ is the number of haplotypes at block *i. j* refers to each haplotype at block *i* and *p*, and *q* represent unique environmental clusters.

### 2.9 Co‐location of previously identified yield and root genes

A selection of well‐researched and mapped barley genes were mapped to Morex V3 to identify those that overlapped with blocks with high LGEI (Mascher et al., 2021). Most of the candidate genes explored were mapped to Morex V3 already (Liu et al., 2024). For all others, GenBank was utilized to obtain sequence data for genes already stored in their system, and the positions of these sequences matched to Morex V3 via the Basic Local Alignment Search Tool of GrainGenes (Yao et al., 2026).

## 3 RESULTS

### 3.1 Genotypic and environmental variation identified across the dataset

The breeding germplasm interrogated through the analyses was found to be moderately diverse with the first two PCs accounting for 29.3% of the variation, which was to be expected as a breeding program dataset. Germplasm formed three groupings as determined by hierarchical analysis (Figure 1). Performance of yield varied across environments (site mean yield 2.5–5.5 t/ha) (Table S3) with additive genetic correlation for yield ranging from −0.08 to 0.92 (Figure 2).

**FIGURE 1:**
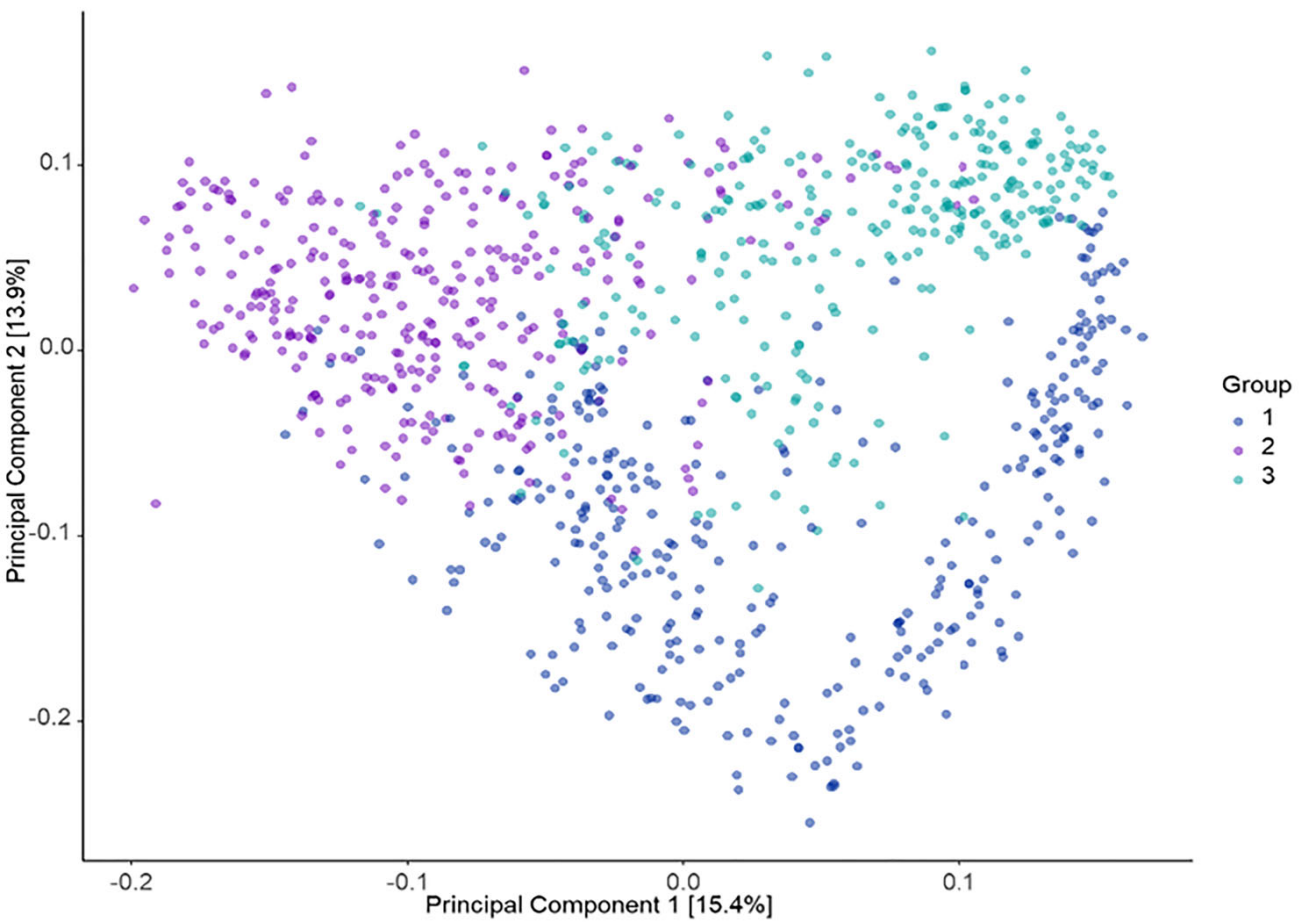
Population structure and diversity of the barley breeding panel (*n* = 961). Principal component analysis based on Roger's distance and calculated using 6599 single nucleotide polymorphisms (SNPs). Groups as determined by hierarchical clustering displayed in different colours as per the key.

**FIGURE 2:**
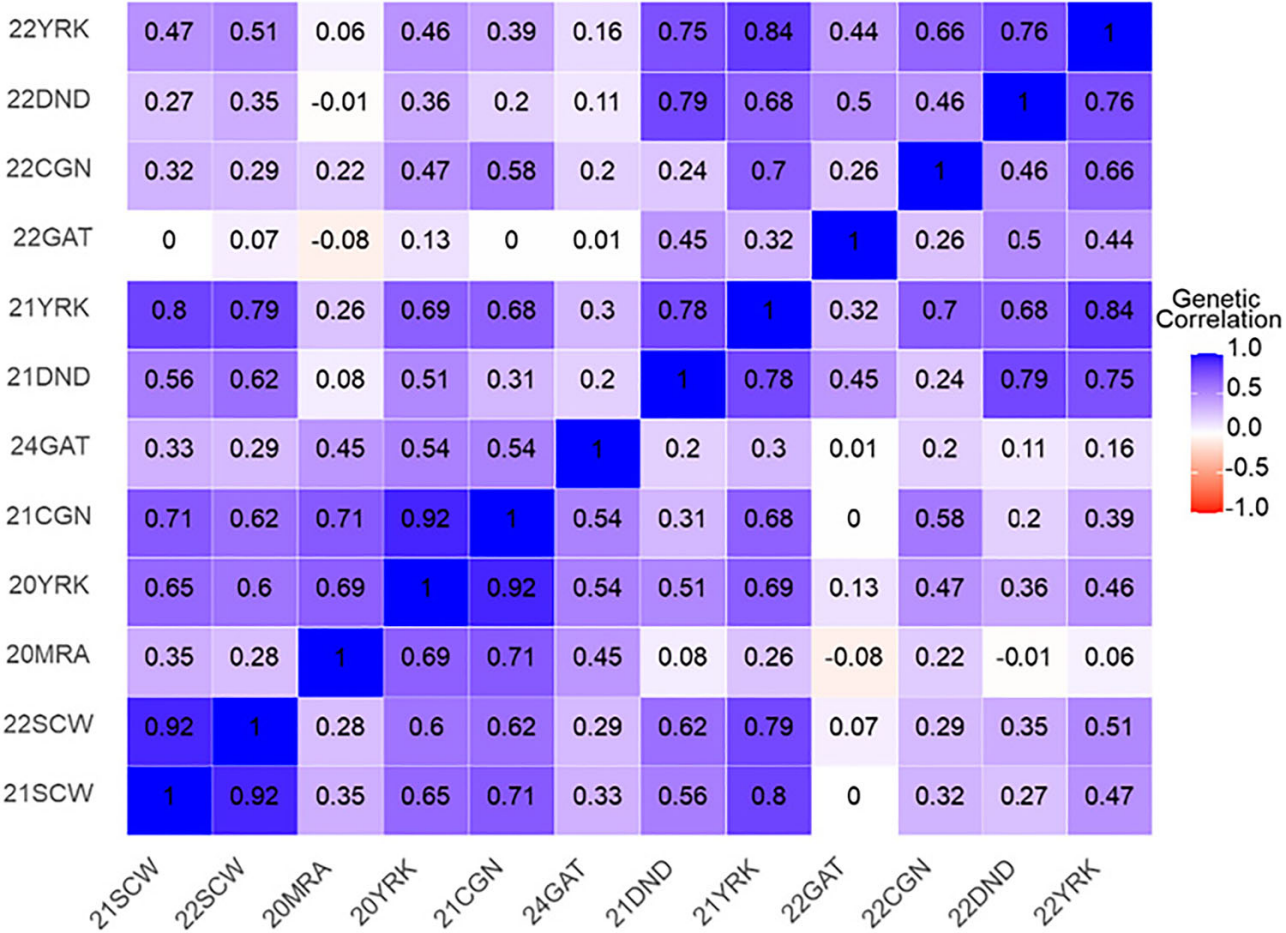
Heat map showing the additive genetic correlation for yield across 12 environments calculated from the factor analytic 4 (FA4) variance structure (Equation 2).

### 3.2 Terms modeling GEIs improve model fit and reveal GEI patterns

Comparing the model statistics for a non‐GEI model and models with iteratively more complex variance structure applied to the GEI term reveals the model‐based benefits of fitting this interaction. The non‐GEI model had the lowest loglikelihood and modeling efficiency and the highest AIC, BIC, and EV%, indicating the model is a poor fit to the dataset. Providing validation of the datasets’ appropriateness to explore GEIs for barley yield. Additionally, all the models without a FA variance structure had an EV% >100%, indicating issues with the ability to fit the data. The FA models all showed the same modeling efficiency, with FA4 also having the greatest loglikelihood and lowest AIC and EV%. The four factors of the FA4 model were found to account for 82% of the genetic variance, and the model showed a statistically significant improvement in likelihood to the FA3 model providing a natural stopping point to prevent overfitting the data with additional factors.

### 3.3 Environmental clusters are characterized by unique and shared haploblocks

The rotated factor loadings for the first three factors from the FA4 model are used to assign the sites to cluster trials (Table 3) (Smith et al., 2021). Four environmental clusters ranging in size from one trial to five trials were formed. Given the latent nature of these loadings, it is difficult to determine the environmental contributions to these clusters. Plotting the second and third factor loadings can provide an illustration of the nature of the clusters and the similarity between loadings (Figure S1). To further validate the FA4 model, the additive genetic correlation from the model was correlated with the outputs from a simplified model with an unstructured variance structure applied to the GEI term. The two matrices were found to have a Mantel correlation of 0.92, indicating the FA4 model is effectively approximating the unstructured variance structure, as per its design.

The haploblock variances as calculated from the haplotype effects specific to each cluster varied; however, there is significant overlap in the top 10% of blocks between clusters (Table S4). Six haploblocks were identified to have high variance for yield across all clusters on chromosomes 2H, 5H, 6H, and 7H. CL1 had the least number of unique top blocks with a single block, b001734, while CL3 and CL4 each had three unique top blocks. Most of the blocks were identified in more than one cluster with 21 blocks being found in two or more analyses. Given the nature of the GBLUP back‐solve approach for marker effects assuming nonzero marker effects, the correlation between block size (number of markers) and block variance was calculated, and no significant correlation was detected in any analysis. The variance of yield haplotype effect also varied across environmental clusters, while CL2 and CL4 both showed a small number of high‐variance blocks, and CL1 and CL3 had a less definitive split between their block variances (Figure 3). To look beyond the top 10% of blocks, a Pearson's correlation for block variance was conducted revealing that CL1 and CL3 were highly correlated (*r* = 0.84) as were CL2 and CL4 (*r* = 0.93) though the correlations between all clusters were ≥0.6 (Figure S2).

**FIGURE 3:**
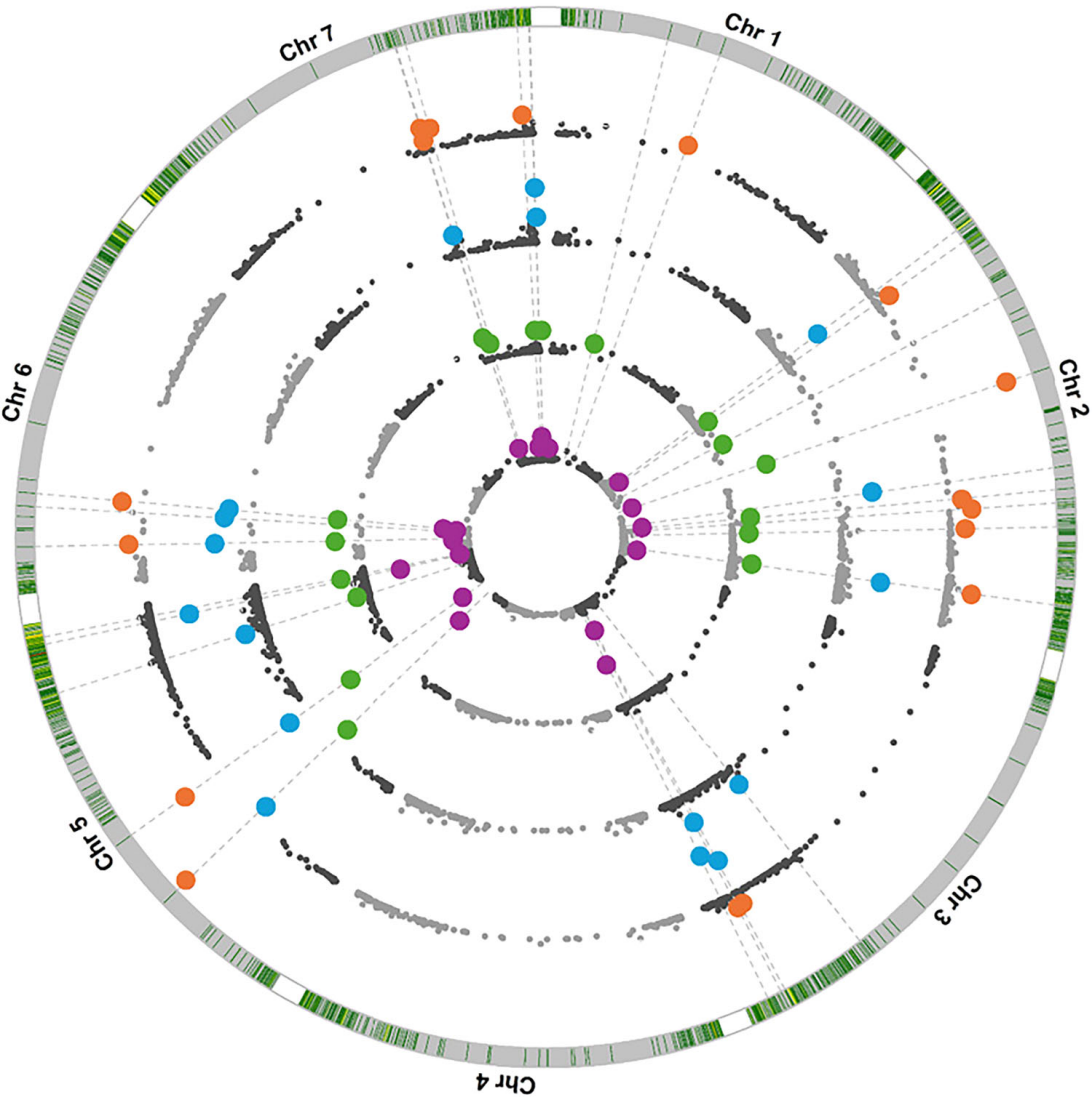
Variance of haplotype effects per block calculated from genomic best linear unbiased predictions (GBLUPs) of each cluster along all seven chromosomes. From inside to outside CL1 (purple), CL2 (green), CL3 (blue), and CL4 (orange). Haplotype effects and block variances calculated as per the local genomic estimated breeding value approach. Block density and positions shown around outside.

Exploration of the diversity and relationship between the block variances for each cluster revealed a large amount of consistent structure with the first two PC alone accounting for over 90% of the variance (Figure 4). Here PC1 was driven by some key high‐variance blocks and had a negative loading for all clusters. PC2 had CL2 and CL4 as negative, while CL1 and CL3 were positive, mimicking the pattern seen in the correlations between block variances. Blocks b000935 and b000936 have negative loadings on PC2 and were top blocks in all analyses, while b001162 and b000626, which were unique to CL1 and CL3, are contributing to positive variation in PC2. Notably, b000287 was a top block for CL2 and CL4 but also CL1 despite its negative loading for PC2.

**FIGURE 4:**
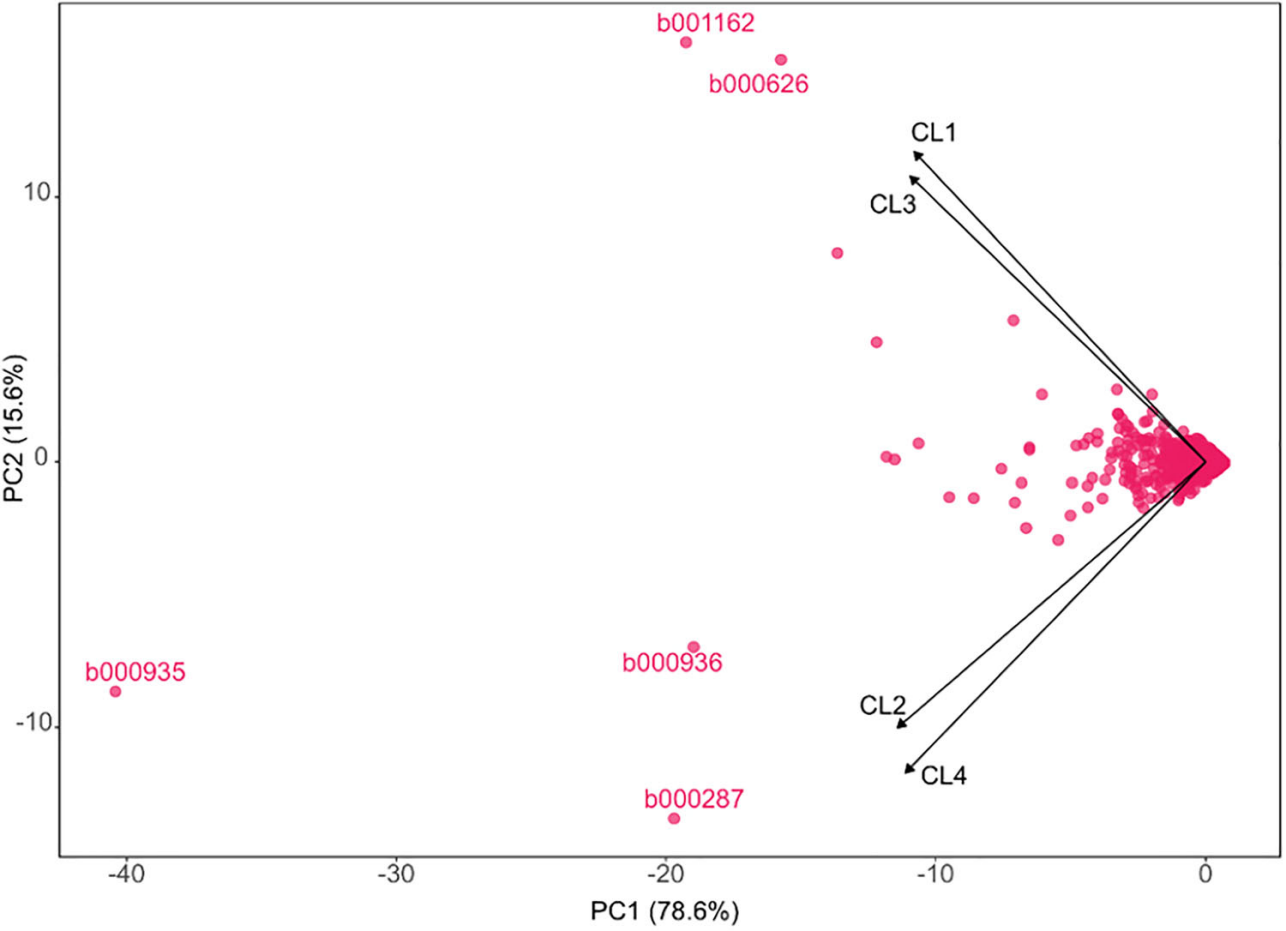
Principal component (PC) analysis of block variances for barley yield at four environmental clusters (CL1–CL4). Blocks represented by pink dots top five blocks driving the PCs are labeled.

### 3.4 Local GEI blocks identified across the genome

The LGEI values for each block identified regions with large changes in haplotype effects across the four environmental clusters on all chromosomes except chromosome 1H (Figure 5). The blocks range vastly in number of SNPs with four of the top 20 LGEI blocks consisting of a single SNP, whereas blocks b000935 and b001604 have over 100 SNPs each (Table S5). The larger blocks are more commonly located toward the center of the chromosome and the smaller blocks toward the ends. Chromosome 2H had six of the top 20 LGEI blocks, while both chromosomes 5H and 6H contained two LGEI blocks that were located directly next to one another.

**FIGURE 5:**
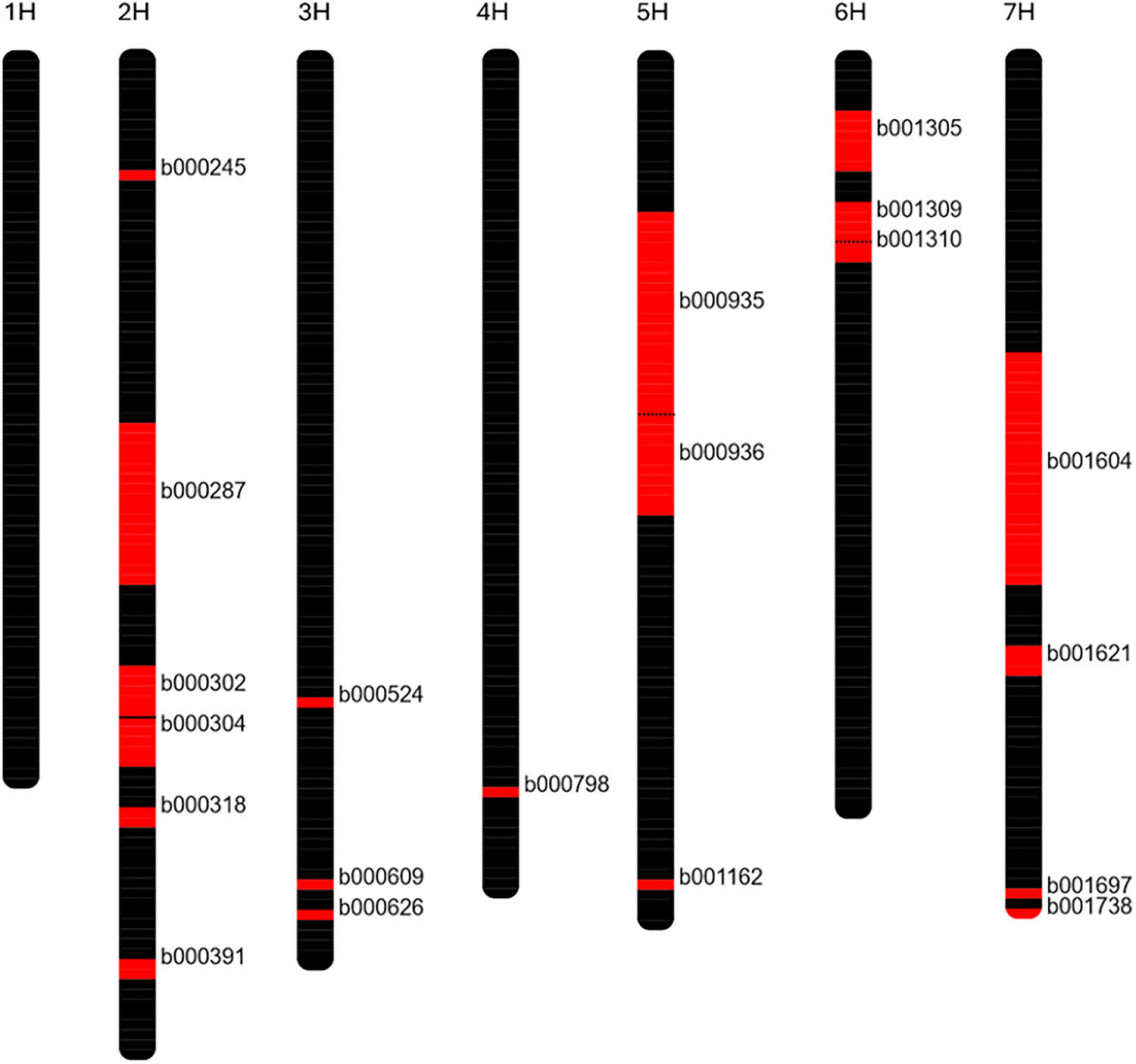
Top 20 blocks with high levels of local genotype‐by‐environment interactions (LGEI) displayed in red on the seven chromosomes of barley (1H–7H). Dashed lines indicate where adjacent blocks are separated.

To further explore the LGEI for yield, the haplotype effects across the four clusters were explored at four of the top 20 LGEI blocks (Figure 6). All four blocks showed large amounts of scale type GEI across all clusters with the effects associated with CL2 often being smaller and with a lower variance than the other clusters. Blocks b000935 (Figure 6B) and b001305 (Figure 6C) both had almost entirely positive effects on yield genomic estimated breeding value. Block b001604 (Figure 6D) had haplotype effects with both positive and negative effects and showed extensive crossover in haplotype effects across the clusters with some haplotypes having a positive effect in one cluster and a negative in another. Block b000304 (Figure 6A) had a predominantly negative effect on yield in CL1, CL2, and CL4 but had many positive haplotypes for CL3. Additionally, the most negative haplotypes in the other three clusters were seen to be those with the most positive effect on yield in CL3. Identifying the specific haplotypes (SNP combination) that were top performing in each cluster for these blocks, we found no cases where the top‐performing haplotype was unique at every cluster (Table S6). The top haplotype at block b000304 was the same at CL1 and CL2, at b000935 was the same at CL1 and CL4, for b001305 was the same at CL1 and CL3, and finally for block b001604, the top‐performing haplotype was the same in CL2 and CL4.

**FIGURE 6:**
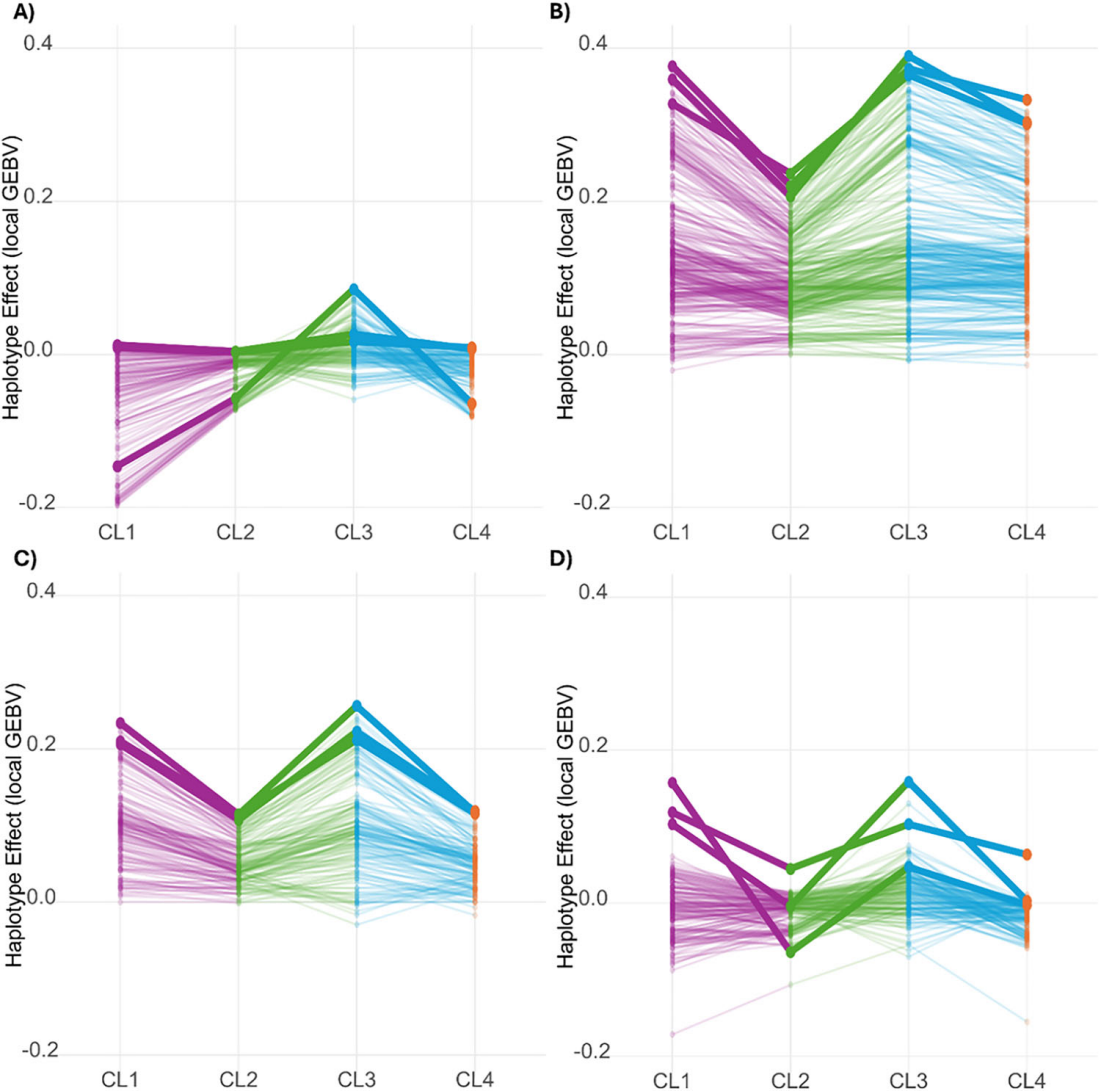
Haplotype effects (local genomic estimated breeding value [GEBV]) across environmental clusters (CL1–CL4) at blocks (A) b000304, (B) b000935, (C) b001305, and (D) b001604. Haplotypes that are the highest effect in at least one cluster are bold.

Co‐locating key barley genes with the LGEI blocks revealed that nine of the LGEI blocks contained genes relevant to barley performance, including root‐related QTL on 2H, 5H, and 6H (Siddiqui et al., 2024) along with multiple resistance QTL (Table S7). Genes affecting yield and its component traits, such as *GPC1*, *HvCO2*, and *qGL2H*, were also identified in key genetic regions influencing GEI for yield (Distelfeld et al., 2008; Mulki & von Korff, 2016; Watt et al., 2020).

## 4 DISCUSSION

This study utilized a range of modern statistical approaches to explore the GEI for yield across a barley breeding panel. GEIs were substantial across the dataset and critical to model explicitly to achieve an adequate fit. Genomic blocks (haploblocks) exhibiting GEI were identified and explored to provide a deeper understanding of the mechanisms of GEI at a haplotype level. Specific haplotypes were then identified as top performing at four key haplotype blocks, demonstrating the practical application of this approach and revealing both consistent and contrasting performance patterns, at the haplotype resolution, across environmental clusters.

### 4.1 FA model with GEI provides the best fit and reveals GEI patterns

It is important to consider the model improvements made by fitting the GEI term in the model in comparison to the non‐GEI model. Environments are rarely independent and will share different proportions of variance with one another. As such, fitting variance structures to consider the relationships between environments is important when characterizing GEIs. This finding is consistent with models for yield in other crops such as wheat, maize, and sorghum (Joshi et al., 2024; Oliveira et al., 2020; Rogers & Holland, 2022). Model terms should not be generalized and will be specific to each new dataset. However, in this barley yield dataset it has been illustrated that the GEI model is a better fit for the data than the non‐GEI model due to improved model statistics (Table 2). The selection of model statistics used to compare the GEI and non‐GEI models was chosen to best represent the model's goodness of fit (EV% and EF), ability to explain the observed data (loglikelihood), balance between these two (AIC and BIC), and the amount of additive genetic variance being captured by the model. It is important, however, to note that there are a large number of alternative model statistics that can be employed to compare models, including broad‐sense heritability, interclass correlation coefficient, and mean absolute error, among others (Falconer, 1996; Koo & Li, 2016). The comparison of GEI models and non‐GEI models using all possible model statistics poses an interesting direction for future research; however, it is beyond the scope of the current study.

**TABLE 2:** Comparison of model statistics for the non‐genotype‐by‐environment interaction (GEI) model and increasingly more complex GEI models. Loglikelihood (Logl), Akaike information criterion (AIC), Bayesian information criterion (BIC), percentage of error variance (EV%), modeling efficiency (EF), and genetic variance captured by the interaction term.

Model	Logl	AIC	BIC	EV%	EF	Genetic variance (%)
Non‐GEI	1941.48	−3758.78	−3313.88	302	0.72	–
Diag	2275.75	−4363.52	−3688.68	105	0.84	40.84
CorV	2762.88	−5331.77	−4635.4	124	0.82	50.28
CorH	2597.76	−5063.52	−4589.7	107	0.83	46.54
FA1	2860.33	−5484.67	−4637.54	88	0.84	51.40
FA2	2954.26	−5628.52	−4623.46	82	0.84	68.10
FA3	2978.88	−5657.77	−4580.91	83	0.84	76.50
FA4	2993.00	−5668.01	−4526.54	81	0.84	82

Abbreviation: FA, factor analytic.

The iClass environmental clustering approach employed in this study was chosen due to its integrated ability to define clusters directly from the model, which yielded four clusters of uneven sizing (Table 3) (Smith et al., 2021). Although iClass was used in this study to illustrate the framework, the approach is not dependent on this specific method; any suitable environmental clustering strategy could be incorporated to define environment groupings within the proposed modeling framework. To further validate the approach of using rotated loading polarity to cluster environments, the genetic correlations from the FA model were contrasted with genetic correlations obtained by fitting a simplified model with unstructured variance structure. As these two correlation matrices were found to be highly correlated, this suggests that the FA model is fulfilling its purpose of approximating an unstructured variance‐covariance model, and hence groupings based on raw genetic correlations would be similar to those obtained by the factor loadings. However, it is noted that when plotting the loadings associated with factors 2 and 3, the magnitude of these factors reveals a potential inconsistency for site 22CGN. Site 22CGN is in CL4, characterized by a positive factor loading at all factors; however, the magnitude of its loading at factor 3 is far greater than the other four sites comprised in this cluster. To our knowledge the magnitude of the iClass loading has not yet been considered when utilizing this approach, but this finding suggests that perhaps it could be considered in some way in the future (Figure S1).

**TABLE 3:** Factor loadings for the first three factors (F1, F2, and F3) for each environment as calculated from the factor analytic 4 (FA4) genotype‐by‐environment interaction (GEI) model. Interaction class (iClass) and environment cluster are determined by polarity of factor loadings.

Trial	F1 loading	F2 loading	F3 loading	iClass	Environmental cluster
21SCW	0.2467	−0.1322	−0.0612	pnn	CL1
22SCW	0.2890	−0.1073	−0.0846	pnn	CL1
20MRA	0.0422	−0.0553	0.0561	pnp	CL2
20YRK	0.1190	−0.0425	0.0616	pnp	CL2
21CGN	0.1518	−0.1003	0.1033	pnp	CL2
24GAT	0.0837	−0.0579	0.0608	pnp	CL2
21DND	0.2080	0.1003	−0.0689	ppn	CL3
21YRK	0.2080	0.0168	0.0156	ppp	CL4
22GAT	0.0686	0.1188	0.0121	ppp	CL4
22CGN	0.1114	0.0334	0.1282	ppp	CL4
22DND	0.2120	0.1957	0.0076	ppp	CL4
22YRK	0.1568	0.0853	0.0219	ppp	CL4

The traces of the shared polarity can be seen in the high correlation in block variance between CL1 and CL3, as well as CL2 and CL4, which share negative and positive loadings at factor 3, respectively. Although factor 3 accounts for less than 10% of the variance, its polarity appears important for understanding the between‐environment GEI for yield (Table 2). This is counterintuitive, as factor 2 accounts for a greater proportion of genetic variance, and one might expect clusters with the same polarity at that factor to be more alike, which is not observed. The importance of factor 3 is further supported by the similar groupings observed in the PC analysis of block variance, as well as by the presence of several high‐variance blocks within these groups (Figure 4; Table S4). One possible explanation relates to the GEI patterns captured by each factor. As discussed earlier, factor 1 reflects overall performance and primarily captures scale‐type GEI, where genotype performance changes in magnitude across environments but rankings remain largely consistent, while subsequent factors are often associated with crossover interactions between sites, where genotype rankings change between environments (Smith et al., 2021). However, some studies suggest that additional factors may also capture scale GEI and that crossover interactions may be better explained using environmental covariates (Tolhurst et al., 2022). While this is beyond the scope of the present study, our results suggest that factor 2 may be capturing scale GEI, whereas factor 3, despite explaining a smaller proportion of genetic variance, appears to be most strongly associated with cross‐over interactions.

### 4.2 GEI exhibited for yield can be dissected using haplotype‐based methodology

Yield in any crop is arguably the “most complex” trait due to its reliance on all other plant processes, including leaf traits, root traits, disease resistance, and nutrient mobility, among many others (Distelfeld et al., 2014; Mühleisen et al., 2014; Smith et al., 1999). As such, barley yield is greatly impacted by changing environmental conditions. When performing separate haplotype analysis, each cluster contained at least one unique high‐variance haploblock showing how the changes in haplotype effects across environments influence the important genetic regions for yield. The six shared high‐variance blocks from all analyses indicate similar regions impacting yield across environments. However, it is important to consider that the interpretation of a block having high variance in different environmental clusters provides no insight into the directionality of the haplotype effects.

This study expands on previous QTL‐by‐environment research by assigning local GEIs at the haplotype block level, allowing a more fine‐tuned approach to explore GEIs than comparisons based on whole‐genotype performance. Previous studies have focused on QTL or marker‐by‐environment interactions, identifying over 100 QTL associated with barley yield, of which only a small proportion appear to be stable across environments (Li et al., 2024; Wang et al., 2016). Disentangling GEI at this higher resolution enables a better understanding of the biological drivers underlying variation in barley yield.

The top 20 LGEI blocks are found throughout the genome and differ in the number of markers from 1 to 185 illustrating that the number of markers is not an influencing factor when considering a haploblock's LGEI (Table S5). Larger blocks were found toward the center of the chromosome and smaller blocks at the ends, which is in line with the LD patterns caused by crossing over during meiosis. Interestingly, two sets of adjacent blocks were identified by the analysis b000935, b000936 and b001309, b001310. This clustering likely reflects shared recombination history and local LD structure rather than functional interaction, although future work could consider how regulatory features or epistatic relationships might be integrated into this framework.

Comparing haplotype effects at four key LEGI blocks across the four clusters reveals some interesting patterns in line with what has already been observed about the environmental clusters (Figure 6). At three of these blocks (b000935, b001305, and b001604), the variance and haplotype effects between CL1 and CL3 are similar as are those between CL2 and CL4, where CL2 and CL4 are often seen to have a smaller range in haplotype effects compared to the other two clusters. However, the haplotype patterns at some of these blocks do reveal the necessity of clustering these sites into four rather than two. Haplotypes at block b000304 show markedly different effects between CL1 and CL3, with the most negative haplotypes in CL1 becoming the most positive in CL3. This pattern suggests the presence of genetic effects that are advantageous under the specific environmental conditions represented by CL3 but not those of CL1 (Figure 6A). While similarities between these cluster pairs have been discussed, crossover GEI between them remains evident, albeit less pronounced than for other cluster comparisons, supporting the need for four distinct environmental groupings. Further validation work is required to establish the consistency of these haplotype effects and GEI patterns across environmental clusters in an independent dataset. However, given the latent nature of this modeling approach, identifying similar environments that would represent the four clusters of this study would pose a challenge.

Haploblocks b00935 and b001305 have clear and stable haplotypes for yield, while their effects change across clusters, and there is little reranking among the top haplotypes providing a clear‐cut breeding objective (Figure 6B,C). Similarly, in block b001604, a top haplotype (b001604.37) with consistently high positive effect for yield was identified as the top performer in CL2 and CL4 and among the top three haplotypes for yield in CL1 and CL4 providing a stable haplotype for yield across all environments (Table S6), while at the same block, the haplotype with the highest effect in CL1 (b001604.134) had a negative effect on yield in both CL2 and CL4. Breeding decisions associated with block b000304 would require deeper understanding of the target environments as the most stable haplotypes have a near‐zero impact on barley yield and hence are an illustration of the trade‐off between stability and performance, especially in the case of CL3 (Figure 6A). Interestingly, despite the strong overall correlations observed between CL1–CL3 and CL2–CL4, the top haplotypes for blocks b000304 and b000935 did not align with these broader cluster relationships. This deviation is consistent with their high GEI values, as blocks with strong local GEI effects may respond to environmental dimensions that differ from those driving the genome‐wide correlation structure. Such patterns illustrate how haplotype‐level analysis can uncover environment‐specific responses that remain obscured when considering only GEI relationships at genotype resolution.

### 4.3 Implications for breeding

While previous studies have focused on the performance of specific genes or QTL across environments, this methodology aims to provide a practical and transferable framework for incorporating GEI into breeding programs. Expanding on existing haplotype‐based approaches, this one‐stage analysis identifies genomic regions that vary in trait response across environments. Although the identification of proposed candidate genes within nine LGEI blocks provides validation of the approach and its robustness, it shifts the focus back to the gene level, which is not the primary objective of this methodology. Additionally, the identified candidate genes cover only a fraction of the potential genes in these blocks that may be contributing to yield. The LGEBV approach applies a breeding framework that considers the effects of groups of markers inherited together, with haplotypes also being more readily tracked within populations than individual markers. Haplotypes can be defined through a variety of approaches, including population‐specific LD thresholds or distance, as their key purpose is to provide “breeder‐friendly” units of inheritance balancing both practicality and fidelity. As demonstrated, this approach enables the identification of both stable haplotypes, such as b001604.37, and haplotypes exhibiting strong GEI, performing favorably in some environments and poorly in others. This information is highly valuable to breeders and can be used to identify individuals carrying desirable haplotypes to inform future crossing decisions, providing a foundation for the targeted deployment of haplotypes in breeding programs.

At present, haplotype‐based breeding is undergoing a period of extensive growth and adoption, yet there is little to no emphasis on the integration of GEI into haplotype‐based approaches. Tools such as “genomicSimulation” can be utilized to optimize breeding programs by identifying optimal crosses based on complementary haplotypes; however, they currently do not consider the impact of GEI when breeding for different environments (Tong et al., 2025; Villiers et al., 2022). GEI‐conscious haplotype‐based analysis can also reveal stable haplotypes in unstable varieties improving the ability to breed for environment stability. Analogously, beneficial haplotypes may be revealed in low‐performing varieties, which can provide greater insight into a variety's genetic potential than a single breeding value. The calculation of these haplotype effects and LGEI measures are closely linked to both the population and environments the analysis is conducted in; to ensure they can provide robust and meaningful value to a breeder, the environments tested need to be representative of the target population of environments. Haplotype frequencies vary greatly; however, a favorable haplotype is rarely in isolation and often groups of haplotypes exhibit the same general trends even if their specific effects are different. This provides more flexibility for breeders interested in utilizing haplotypes to not restrict their decisions to a specific haplotype but rather the group of “favorable” haplotypes. The LGEBV approach has also been discussed in the context of combining all the top haplotypes for a trait to create the “ultimate genotype,” one barrier to which is the knowledge that due to GEI the beneficial haplotypes are not the same across environments (Hayes et al., 2024). This methodology serves as a starting point for addressing these issues in haplotype‐based breeding as breeders can identify the top‐performing haplotypes specific to their target population of environments or select for stability as per their unique breeding objectives.

Beyond LGEI haplotypes, this methodology can help breeders understand the environments in their trial data more deeply through both shared genetic controls and patterns of GEI as seen through the similarities between CL2 and CL4 (Figure S1). However, the methodology is restricted through the use of only latent variables to characterize GEI and define the clusters with explicit environmental data incorporated in the model or clustering process. Some research has suggested that the FA latent variables do not account for all the crossover GEI present between environments (Piepho & Blancon, 2023; Tolhurst et al., 2022), potentially explaining the apparent greater importance of factor 3 compared to factor 2 when correlating block variances and comparing haplotype effects. Future work could also integrate environmental covariates into this framework to enhance the resolution of environmental clustering and better identify the specific environmental drivers contributing to GEIs. An environmental covariate framework would allow a biological interpretation of favorable haplotypes, more informative candidate gene identification, and more actionable information for breeders on haplotype GEI. In addition, iClasses cannot be predicted and are rarely consistent changing as more environments are added to the model due to new factors being defined to explain the variation. However, some success has been had in identifying the environmental patterns in iClasses through correlating environmental variables with factor loadings, allowing for prediction into the future based on expected iClass frequencies (Fradgley et al., 2025). Determining the frequencies of each environmental cluster would allow breeders to make more informed decisions regarding which haplotypes are most favorable when no clear stable haplotype is present in the population. This framework is transferable to other crops and with relatively simplistic implementation and easily interpretable outcomes making it desirable for implementation in breeding programs.

## 5 CONCLUSION

This large, complex multi‐environment barley yield dataset exhibited extensive GEI, which was best captured using an FA4 variance structure. Across environments, we identified genomic regions (haploblocks) contributing to yield, alongside many regions that were consistently important across the four environmental clusters. Many of these blocks also exhibited strong GEI, with specific haplotypes showing a wide range of effects across clusters. Further analysis of four key blocks identified both stable haplotypes for yield and haplotypes with large positive effects in a single cluster but clear trade‐offs in others. Together, these results demonstrate that this methodology provides a framework for exploring GEI at haplotype resolution and for identifying target haplotypes with stable or environment‐specific performance to support breeding objectives.

## AUTHOR CONTRIBUTIONS

**Stephanie M. Brunner**: Conceptualization; data curation; formal analysis; investigation; methodology; validation; visualization; writing—original draft. **Zachary Aldiss**: Formal analysis; writing—review and editing. **Samir Alahmad**: Data curation; writing—review and editing. **Madhav Pandit**: Formal analysis; writing—review and editing. **Silvina Baraibar**: Resources; writing—review and editing. **Dini Ganesalingam**: Resources; writing—review and editing. **David Moody**: Resources; writing—review and editing. **Lee Hickey**: Resources; supervision; writing—review and editing. **Kai P. Voss‐Fels**: Conceptualization; writing—review and editing. **Hannah Robinson**: Conceptualization; formal analysis; supervision; writing—review and editing.

## CONFLICT OF INTEREST STATEMENT

The authors declare no conflicts of interest.

## Supporting information




**Supplementary Figure 1**. Rotated factor loadings of second and first factor for each site from FA4 GBLUP model.
**Supplementary Figure 2**. Pearsons' correlation in block variance for yield LGEBV between environmental clusters.

**Supplementary Table 1**. Genotype concurrence between 12 experimental sites.
**Supplementary Table 2**. Number of SNPs and alleles per block, block variance in each environmental cluster.
Supplementary Table 3. Summary statistics for each trial.
**Supplementary Table 4**. Top 10% of haploblocks by variance identified at each cluster.
**Supplementary Table 5**. Positions of top 20 local GEI blocks as shown in Figure 5.
**Supplementary Table 6**. All haplotypes and sequences with effects across each cluster for haplotypes shown in Figure 6.
**Supplementary Table 7**. QTL identified in top 20 LGEI blocks and their functions.

## Data Availability

The data that support the findings of this study are available from InterGrain Pty Ltd, but restrictions apply to the availability of these data, which were used under license for the current study and so are not publicly available. Data are, however, available from the authors upon reasonable request and with permission of InterGrain Pty Ltd. Code for the analysis pipeline from initial MET model fitting to haplotype‐based analysis with HapSelect are provided at https://doi.org/10.5281/zenodo.21270634; Brunner (2026). Further, the data were not made available during the peer‐review process due to their sensitive and proprietary nature.
