# Supplementary material for: A haplotype‐based approach to explore genotype‐by‐environment interactions for barley yield
Source: Plant Genome. 2026 Sep 29;19(4):e70310. doi: 10.1002/tpg2.70310 (PMC13621458; doi:10.1002/tpg2.70310)
Supplement: Supplementary file 1 [file TPG2-19-e70310-s002.docx]

**A)**

*Supplementary Figure 1.A) Rotated factor loadings of first and second factor for each site from FA4 GBLUP model. B) Rotated factor loadings of the second and third factors for each site from FA4 GBLUP model. Coloured by environmental cluster as determined by polarity of factor loadings.*

**B)**

*Supplementary Figure 2. Pearsons’s correlation in block variance for yield LGEBV between environmental clusters.*
